# Rho-kinase inhibitor coupled to peptide-modified albumin carrier reduces portal pressure and increases renal perfusion in cirrhotic rats

**DOI:** 10.1038/s41598-019-38678-5

**Published:** 2019-02-19

**Authors:** Sabine Klein, Franziska Frohn, Fernando Magdaleno, Catharina Reker-Smit, Robert Schierwagen, Irela Schierwagen, Frank Erhard Uschner, Fransien van Dijk, Dieter O. Fürst, Sonja Djudjaj, Peter Boor, Klaas Poelstra, Leonie Beljaars, Jonel Trebicka

**Affiliations:** 1 Department of Internal Medicine I, University of Bonn, Bonn, Germany; 2 Department of Internal Medicine I, Goethe University Frankfurt, Frankfurt, Germany; 3 Department of Pharmacokinetics, Toxicology and Targeting, Groningen Research Institute for Pharmacy, University of Groningen, Groningen, Netherlands; 4 Institute for Cell Biology, Department of Molecular Cell Biology, University of Bonn, Bonn, Germany; 5 Institute of Pathology, RWTH University of Aachen, Aachen, Germany; 6 Division of Nephrology, RWTH University of Aachen, Aachen, Germany; 7 European Foundation for the Study of Chronic Liver Failure – EF Clif, Barcelona, Spain; 8 Department of Medical Gastroenterology and Hepatology, Odense University Hospital, Odense, Denmark; 9 Institute for Bioengineering of Catalonia, Barcelona, Spain

## Abstract

Rho-kinase (ROCK) activation in hepatic stellate cells (HSC) is a key mechanism promoting liver fibrosis and portal hypertension (PTH). Specific delivery of ROCK-inhibitor Y-27632 (Y27) to HSC targeting mannose-6-phosphate-receptors reduces portal pressure and fibrogenesis. In decompensated cirrhosis, presence of ascites is associated with reduced renal perfusion. Since in cirrhosis, platelet-derived growth factor receptor beta (PDGFRβ) is upregulated in the liver as well as the kidney, this study coupled Y27 to human serum albumin (HSA) substituted with PDGFRβ-recognizing peptides (pPB), and investigated its effect on PTH in cirrhotic rats. *In vitro* collagen contraction assays tested biological activity on LX2 cells. Hemodynamics were analyzed in BDL and CCl_4_ cirrhotic rats 3 h, 6 h and 24 h after i.v. administration of Y27pPBHSA (0.5/1 mg/kg b.w). Phosphorylation of moesin and myosin light chain (MLC) assessed ROCK activity in liver, femoral muscle, mesenteric artery, kidney and heart. Three Y27 molecules were coupled to pPBHSA as confirmed by HPLC/MS, which was sufficient to relax LX2 cells. *In vivo*, Y27pPBHSA-treated rats exhibited lower portal pressure, hepatic vascular resistance without effect on systemic vascular resistance, but a tendency towards lower cardiac output compared to non-treated cirrhotic rats. Y27pPBHSA reduced intrahepatic resistance by reduction of phosphorylation of moesin and MLC in Y27pPBHSA-treated cirrhotic rats. Y27pPBHSA was found in the liver of rats up to 6 hours after its injection, in the HSC demonstrated by double-immunostainings. Interestingly, Y27pPBHSA increased renal arterial flow over time combined with an antifibrotic effect as shown by decreased renal *acta2* and c*ol1a1* mRNA expression. Therefore, targeting the ROCK inhibitor Y27 to PDGFRβ decreases portal pressure with potential beneficial effects in the kidney. This unique approach should be tested in human cirrhosis.

## Introduction

In liver cirrhosis, portal hypertension (PHT) is caused by increased intrahepatic vascular resistance to portal blood flow, partially due to contraction and increased collagen deposition by hepatic stellate cells (HSC), the dominant cells contributing to liver fibrosis^1^. Together with decreased systemic and splanchnic resistance, these factors lead to PHT, the major driver for most of the clinical complications associated with cirrhosis. Presence of ascites, in particular, is associated with a worse outcome, while ascites itself is at least partly due to decreased renal perfusion^2^.

Activated HSC not only synthesize extracellular matrix (ECM) components, but are also the primary profibrotic cells, participating in the regulation of liver microcirculation and PTH^3,4^. Among other factors, such as PDGFRβ, overactivation of ROCK is a core feature of HSC activation^5–7^. Thus, inhibition of ROCK attenuates liver fibrosis and the associated development of PTH^8–10^. Nevertheless, there is the paradox of increased RhoA/ROCK expression and activity within the liver and decreased expression outside the liver (i.e. splanchnic vessels) contributing partially to the observed hypocontractility and vascular dilatation in cirrhosis^11–13^. This finding is specific for liver cirrhosis, since there are recent reports demonstrating that ROCK is overactivated in mesenteric vessels of aged animals^14^, however, the opposite is the case in liver cirrhosis^15–17^. Also in other cardiovascular pathologies mesenteric vascular tone is increased^18^, while during cirrhosis with portal hypertension in splanchnic and mesenteric vessels ROCK activity is blunted^15–17^. Hence, a decrease in mean arterial pressure using systemic ROCK inhibition by Y-27632 (Y27) might further decrease renal perfusion. Therefore, targeting of Y27 specifically to the liver and the kidney leading to intrahepatic and intrarenal vasodilation would decrease portal pressure and improve renal function.

Previous work demonstrated that specific ROCK inhibitors, such as Y27 delivery to the Mannnose-6-phosphate-Insulin-like Growth Factor II (M6P-IGFII) receptor, decreased portal pressure^19^. However, PDGFRβ is not only increased in the liver^20,21^, but, also in the kidneys, especially in kidney injury^22,23^. Therefore, this study investigated the time- and dose-dependent effect of Y27 with HSA modified with PDGFRβ-recognizing peptides (Y27pPBHSA) on portal hypertension and renal perfusion in cirrhotic rats.

## Results

### Three Y27 molecules coupled to pPBHSA are sufficient to relax LX2 cells *in vitro*

**Figure 1:**
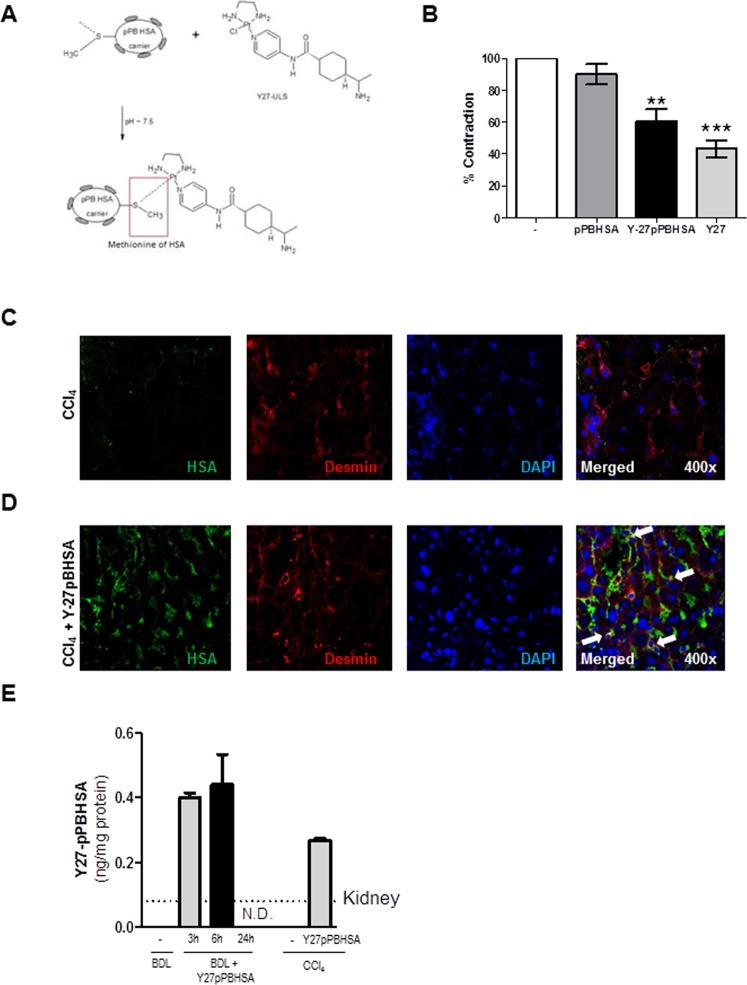
Three Y27 molecules coupled to pPBHSA are sufficient to relax LX2 cells *in vitro*. MALDI-TOF mass spectrometry determined the molecular weight of Y27pPBHSA = 77693 Da, and HPLC confirmed 3.0 molecules of Y27 coupled to pPBHSA. (**A**) LX2 cells were treated for 72 h with 3.3 μM of Y27-pPBHSA (construct containing three molecules of Y27) and 10 μM of Y27 (which is equal to the amount of Y27 in the batch of Y27-pPBHSA). The same amount of pPBHSA was used as in Y27pPBHSA-treated LX2 cells. Results are expressed as mean ± standard error of the mean (SEM); n = 4/group, **p < 0.01 and ***p < 0.001 for treated vs. non-treated LX2 cells. (**B**) Y27pPBHSA targets HSC in cirrhotic rats. Immunofluorescence in frozen sections of cirrhotic livers shows absent co-localization of human serum albumin (HAS, green) with desmin, a marker of HSC (red), in CCl_4_- cirrhotic rats (**C**) but co-localization in Y27pPBHSA-treated cirrhotic rats. (**D**) pPBHSA ELISA in Y27pPBHSA-treated cirrhotic rats expressed as ng/mg liver-protein or kidney-protein. (**E**) Abbreviations: HSA, human serum albumin; BDL, bile duct ligation; ND, not detected.

To optimize the therapeutic potential of Y27, Y-27632 molecules were coupled to pPBHSA at a ratio of 3:1 as assessed by MALDI-TOF mass spectrometry and HPLC analysis. The compound had a molecular weight of 77.396 kDa (Fig. 1A). Functional *in vitro* experiments were performed on LX2 cells (human HSC cell line) in order to assess biological activity of the conjugated Y27. Cells were treated with the carrier alone, the ROCK inhibitor Y27 or with Y27pPBHSA for 72 h. The construct containing three molecules of Y27 relaxed LX2 by 40% as shown by the percentage of collagen gel contraction compared to controls (contraction index control = 100 ± 0.0%; Y27-unconjugated = 43.5 ± 5.3%; Y27pPBHSA = 60.7 ± 7.4%) (Fig. 1B). As previously shown by the release kinetics^24^, the modified Y27 with targeted carrier retained its biological activity due to minimal modification and mild chemical conditions, and the ROCK-inhibitory effects are most likely due to the intracellular release of Y27 from the internalized construct, which is then degraded in the cells.

### Y27pPBHSA targets HSC in cirrhotic rats

Previously, biodistribution studies of the complex have shown that 48% of pPBHSA is taken up by the liver within 10 min after injection, while 34% is still present in the blood compartment, and 18% in kidney^20^. Similarly, Y27 concentrations in whole livers of animals receiving an Y27-conjugate were much higher, while in the systemic circulation Y27-conjugates disappeared after 24 hours^5^.

To verify *in vivo* the specific delivery of Y27 to HSC, co-localization studies were carried out using specific markers for HSC (desmin, cytoplasmic) and antibody against HSA, which in the rat liver recognizes only the construct. The major part of pPBHSA was localized in HSC as shown by co-localization in cryostat sections of cirrhotic rats (Fig. 1C,D). Importantly, the pPBHSA ELISA result demonstrate that the drug is primarily up-taken in the liver (compared to the kidney accumulation shown as dashed line) and is detectable 3 hours after injection in both models of liver cirrhosis and is up to 6 hours after injection (Fig. 1E). Taken together, these results demonstrate the specific delivery of the carrier of Y27 into the HSC of Y27pPBHSA-treated compared to non-treated rats.

### Y27pPBHSA lowers portal pressure and hepatic vascular resistance without systemic hemodynamic changes in cirrhotic rats

To investigate whether Y27pPBHSA modifies portal and systemic hemodynamics, *in vivo* dose- and time-dependent experiments were conducted. To establish the most effective dose, two different single doses of Y27pPBHSA (i.v. 0.5 and 1 mg/kg b.w.) were analyzed after 3 h, 6 h and 24 h in bile duct ligated (BDL) cirrhotic rats and after 3 h in CCl_4_ cirrhotic rats.

**Figure 2:**
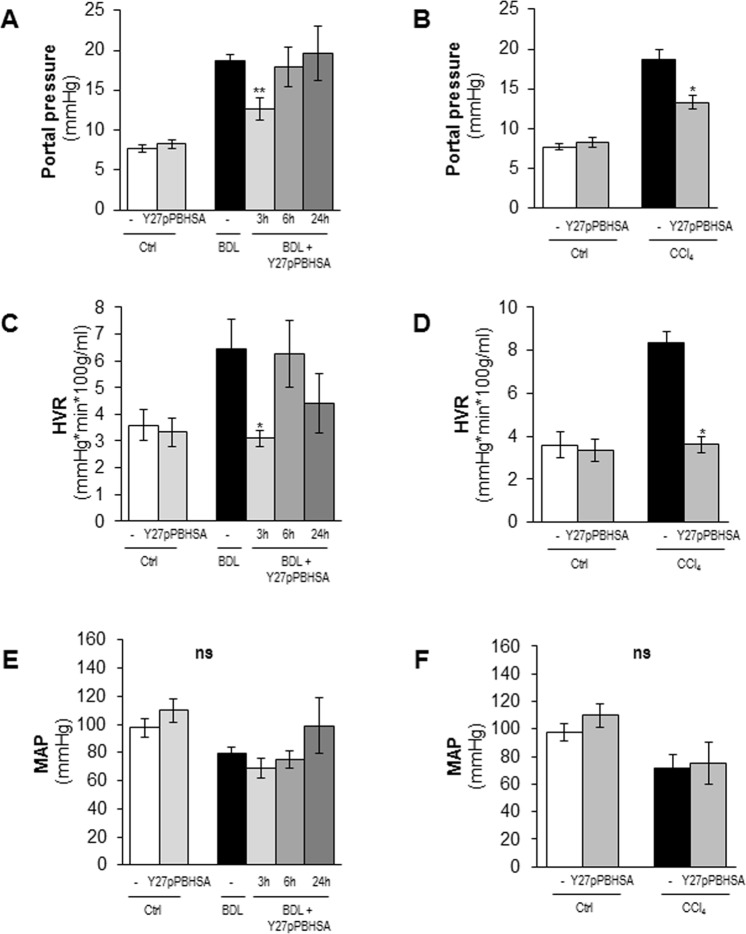
Y27pPBHSA lowers portal pressure and hepatic vascular resistance without systemic effects in cirrhotic rats. Systemic and portal hemodynamics were analyzed in hypertensive BDL and CCl_4_ cirrhotic rats using a single i.v. administration of Y27pPBHSA (1 mg/kg b.w) after 3 h, 6 h and 24 h. Portal pressure (**A**,**B**), hepatic vascular resistance (HVR) (**C**,**D**) and mean arterial pressure (MAP) (**E**,**F**) were investigated. Results are expressed as mean ± standard error of the mean (SEM); n = 6/group, *p < 0.05 and **p < 0.01 for Y27-pPBHSA-treated *vs*. non-treated cirrhotic rats. Abbreviations: HVR, hepatic vascular resistance; MAP, mean arterial pressure; BDL, bile duct ligation.

As anticipated, BDL and CCl_4_ cirrhotic rats had significantly increased PP (Fig. 2A,B) and subsequently increased hepatic vascular resistance (HVR) (Fig. 2C,D) and hepatic arterial flow (Suppl. Table S3). Mean arterial pressure (MAP) (Fig. 2E,F), systemic vascular resistance (SVR), splanchnic vascular resistance (SpVR), and cardiac output (Suppl. Table S3) were decreased in non-treated cirrhotic animals compared to non-cirrhotic control animals, confirming the presence of hyperdynamic circulation in our models.

A dose of 0.5 mg/kg Y27pPBHSA showed no significant portal pressure lowering effect (Suppl. Table S3) and no effects on Sirius red staining and α-smooth muscle actin (αSMA) protein expression compared to non-treated BDL cirrhotic rats (Suppl. Fig. 1). By contrast, a dose of 1 mg/kg Y27pPBHSA reduced PP by 33% (Fig. 2A) and HVR by 57% (Fig. 2C) in Y27pPBHSA-treated animals compared to non-treated BDL cirrhotic rats three hours after injection. These effects were not observed after 6 h and 24 h (Fig. 2A,C). These hemodynamic effects were confirmed in CCl_4_-induced cirrhosis using 1 mg/kg Y27pPBHSA three hours after injection (Fig. 2B,D).

Importantly, no statistically significant changes on MAP (Fig. 2E,F), SVR, SpVR and cardiac output were observed in Y27pPBHSA-treated cirrhotic rats (Suppl. Table S3).

### Hepatic and extrahepatic effects of Y27pPBHSA in cirrhotic rats

Hepatic and extrahepatic toxicity was analyzed in rat liver sections using hematoxylin and eosin (H&E) staining, and by measurement of aminotransferases (ALT, AST), bilirubin, blood urea nitrogen (BUN), C-reactive protein (CRP) and creatine kinase (CK) in serum of investigated animals. There was no indication for liver or extrahepatic toxicity of Y27pPBHSA in the cirrhotic animals (Suppl. Table S4 and Suppl. Fig. 2).

Since ROCK phosphorylates Ser19 of MLC^25^ and Thr558 of moesin to regulate the assembly of stress fibers and cell contraction^26^, we analyzed the effect of Y27pPBHSA on the phosphorylation of MLC^Thr18/Ser19^ and moesin^Thr558^ in liver, kidney, heart, mesenteric artery and muscle, as a readout of ROCK activity.

**Figure 3:**
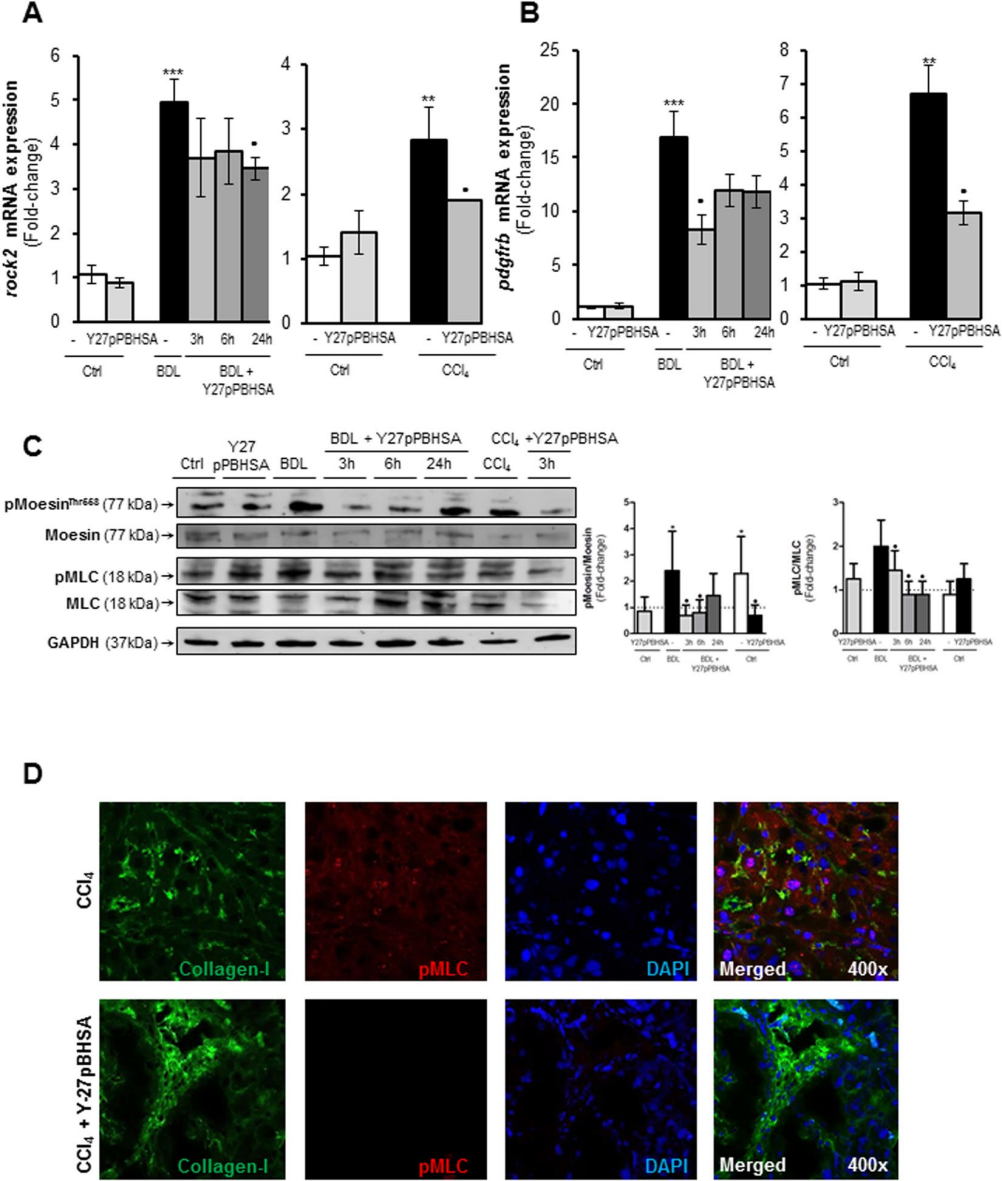
Y27pPBHSA reduces hepatic rock2 and pdfgrb mRNA expression and phosphorylation of moesin and MLC in cirrhotic rats. Hepatic *rock2* and *pdgfrb* mRNA expression in Y27pPBHSA (1 mg/kg)-treated cirrhotic rats. Results are expressed as mean ± standard error of the mean (SEM); n = 6/group, **p < 0.01 and ***p < 0.001 for BDL or CCl_4_ cirrhotic vs. corresponding control rats, and ^•^*p* < 0.05 for Y27pPBHSA-treated vs. non-treated BDL or CCl_4_ cirrhotic rats. (**A**,**B**) Western blots for p-moesin/moesin, p-MLC/MLC and GAPDH were performed from liver of cirrhotic rats treated for 3 h, 6 h and 24 h with Y27pPBHSA (1 mg/kg). Results are expressed as mean ± standard error of the mean (SEM); n = 6/group, *p < 0.05 for BDL cirrhotic vs. corresponding control rats; ^•^*p* < 0.05 for Y27pPBHSA-treated vs. non-treated cirrhotic rats. (**C**) Immunofluorescence in frozen sections of cirrhotic liver shows collagen (green) and p-MLC (red) expressions in septa of Y27pPBHSA-treated cirrhotic rats. (**D**) Abbreviations: p-MLC, phospho-myosin light chain; BDL, bile duct ligation.

In line with the *in vivo* hemodynamics studies, significant reduction of hepatic *rock2* and *pdgfrb* mRNA expression (Fig. 3A,B) and decreased p-moesin and p-MLC were found after injection of Y27pPBHSA in BDL and CCl_4_ cirrhotic rats (Fig. 3C). As expected, co-localization studies in cryostat liver sections showed a reduction of collagen and p-MLC expression in the fibrotic septa of Y27pPBHSA-treated cirrhotic rats (Fig. 3D), confirming the observed effect of the HSC-directed ROCK inhibitor.

**Figure 4:**
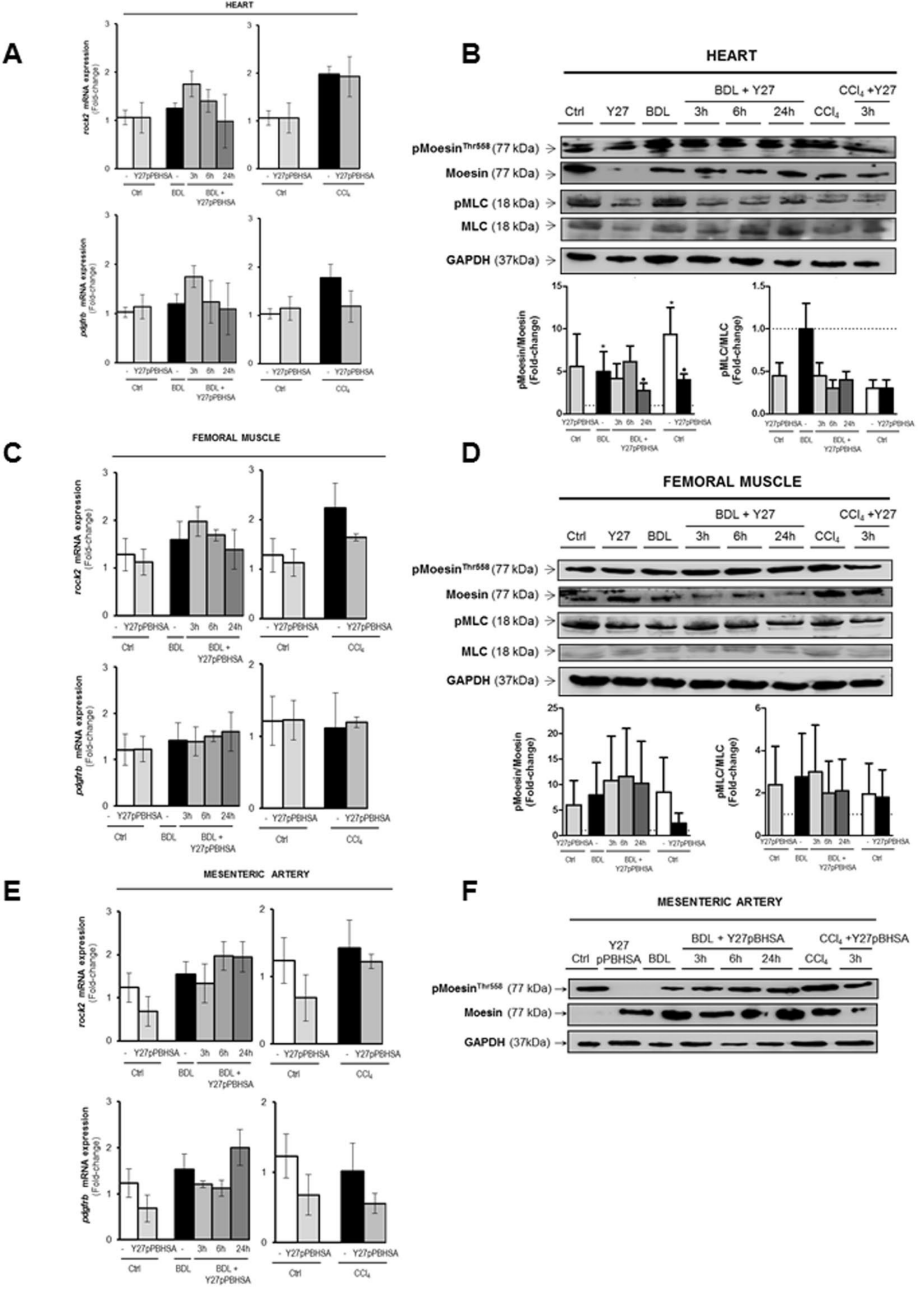
Extrahepatic effects of Y27pPBHSA in cirrhotic rats. Cardiac *rock2* and *pdgfrb* mRNA expression in Y27pPBHSA-treated cirrhotic rats. (**A**) Western blots for p-moesin/moesin, p-MLC/MLC and GAPDH in the heart from cirrhotic rats treated for 3–24 h with Y27pPBHSA. (**B**) *rock2* and *pdgfrb* mRNA expression in femoral muscle of Y27pPBHSA-treated cirrhotic rats (**C**). Western blots for p-moesin/moesin, p-MLC/MLC and GAPDH in femoral muscle from cirrhotic rats treated for 3–24 h with Y27pPBHSA. (**D**) *rock2* and *pdgfrb* mRNA expression in mesenteric artery of Y27pPBHSA-treated cirrhotic rats. (**E**) Western blots for p-moesin/moesin and GAPDH in mesenteric artery from cirrhotic rats treated for 3–24 h with Y27pPBHSA. (**F**) Results are expressed as mean ± standard error of the mean (SEM); n = 6/group. Abbreviations: p-MLC, phospho-myosin light chain; BDL, bile duct ligation.

A decrease in cardiac p-moesin and p-MLC, associated with slightly increased *rock2* and *pdgfrb* mRNA expressions was observed in Y27pPBHSA-treated animals compared to non-treated BDL cirrhotic rats (Fig. 4A,B). This effect was less pronounced in CCl_4_ animals (Fig. 4A,B). In femoral muscle, *rock2* and *pdgfrb* mRNA expression as well as p-moesin and p-MLC remained unchanged after Y27pPBHSA treatment (Fig. 4C,D). Similarly, *rock2* and *pdgfrb* mRNA expression and p-moesin remained unchanged in the mesenteric artery of Y27pPBHSA-treated BDL and CCl_4_ cirrhotic rats when compared to non-treated animals (Fig. 4E,F).

Taken together, these results suggest that targeted ROCK inhibition in HSC hampers hepatic MLC and moesin phosphorylation as well as hepatic *rock2* and *pdgfrb* mRNA expression, which could be the result of the observed reduced HSC contraction and PP in cirrhotic rats without systemic hemodynamic effects.

### Y27pPBHSA improves renal hemodynamics in cirrhotic rats

**Figure 5:**
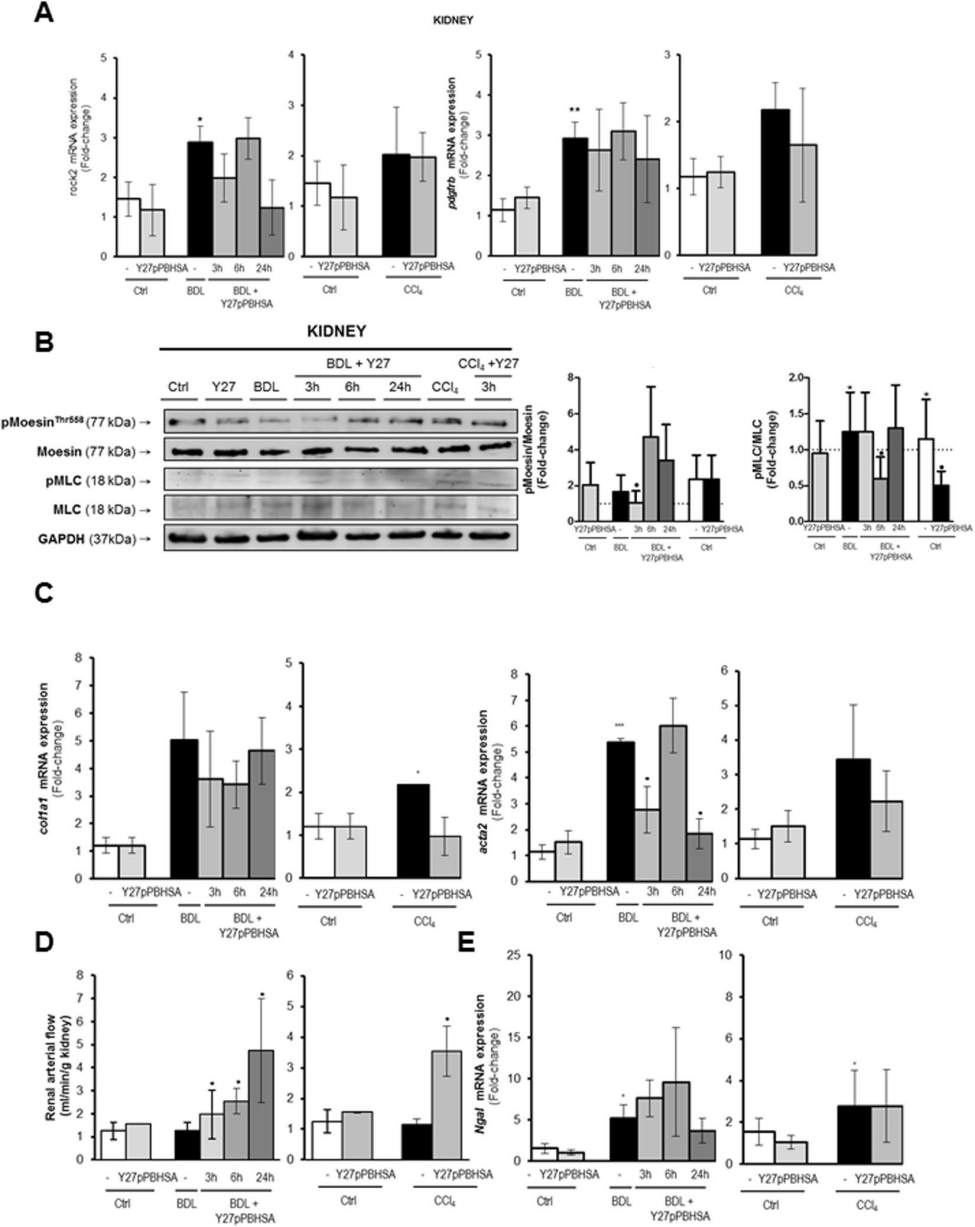
Y27pPBHSA improves renal hemodynamics in cirrhotic rats. Renal *rock2* and *pdgfrb* mRNA expression. (**A**) Western blots for p-moesin/moesin, p-MLC/MLC and GAPDH. (**B**) *col1a1* and *acta2* mRNA expression. (**C**) Renal arterial flow in ml/min/kidney (**D**) and *ngal* mRNA expression (**E**) in kidney from Y27pPBHSA-treated BDL or CCl_4_ cirrhotic rats. Results are expressed as mean ± standard error of the mean (SEM); n = 6/group, *p < 0.05, **p < 0.01 and ***p < 0.001 for BDL or CCl_4_ cirrhotic rats vs. corresponding control rats; ^•^*p* < 0.05 for Y27pPBHSA-treated vs. non-treated cirrhotic rats. Abbreviations: p-MLC, phospho-myosin light chain; BDL, bile duct ligation: Ngal, neutrophil gelatinase-associated lipocalin.

Compared to non-cirrhotic rats, both *rock2* and *pdgfrb* mRNA expression were significantly upregulated in kidneys of BDL cirrhotic rats, while in CCl_4_ cirrhotic animals, this trend was not statistically significant (Fig. 5A). Y27pPBHSA treatment had no significant effect on renal *rock2* and *pdfrb* mRNA expression (Fig. 5A), but it decreased p-moesin and p-MLC compared to non-treated cirrhotic rats (Fig. 5B). Moreover, *col1a1* and *acta2* mRNA expression showed a tendency towards decrease in the Y27pPBHSA-treated cirrhotic rats compared to non-treated cirrhotic rats (Fig. 5C). After already 3 h, Y27pBHSA-treated rats in the BDL and the CCl_4_ model had increased renal arterial flow, with more prominent effects after 24 h (Fig. 5D).

By contrast, *ngal* mRNA expression (neutrophil gelatinase-associated lipocalin), a marker of renal dysfunction^27,28^, was markedly increased in BDL and CCl_4_ cirrhotic rats confirming the presence of kidney damage by BDL and CCl_4_ insults, but remained unchanged by Y27pPBHSA treatment (Fig. 5E). Similarly, serum creatinine and sodium levels remained unchanged (Suppl. Table S4).

### Y27pPBHSA treatment and hepatic and extrahepatic fibrogenesis

As expected, hepatic HP content and Sirius red staining showed no difference in fibrosis after Y27pPBHSA injection (Suppl. Fig. 3A–C). Yet, *col1a1* mRNA expression was reduced 3 h after Y27pPBHSA treatment in BDL and CCl_4_ cirrhotic rats (Suppl. Fig. 3D,E). No changes in *col1a1* mRNA expression were observed in the heart, the mesenteric artery and the femoral muscle in Y-27pPBHSA-treated BDL and CCl_4_ cirrhotic rats (data not shown).

αSMA protein (Suppl. Fig. 4A) and hepatic *acta2* mRNA expression were similar in Y27pPBHSA-treated BDL cirrhotic rats and had a tendency towards a decrease in CCl_4_ cirrhotic rats (Suppl. Fig. 4B,C), while no changes in *acta2* mRNA expression were observed in the heart, the mesenteric artery and the femoral muscle in Y27pPBHSA-treated BDL and CCl_4_ cirrhotic rats (data not shown).

## Discussion

The present study shows that ROCK inhibition targeted to PDGFRβ might be a promising strategy to treat PHT in cirrhosis without adverse extrahepatic hemodynamic effects. Moreover, the study demonstrated for the first time that renal ROCK activation occurs in cirrhotic kidneys. The selective delivery of a single dose of Y27pPBHSA efficiently lowered PP and improved renal perfusion in two different models of liver cirrhosis, without systemic hypotensive effects.

ROCK activation in HSC is a critical mechanism of cellular fibrogenesis^29^, contractility^30^, proliferation^31^ and fate^32^, and it represents an attractive target to treat liver fibrosis and PTH^29,33^. Previously, different groups have suggested that targeting ROCK in HSC was a good approach to decrease fibrosis and PTH^10,19,34^. However, systemic administration of ROCK inhibitors (e.g. Y27) leads to extensive systemic effects^11^. Nevertheless, when using targeting strategies to M6P receptor, Y27 did not elicit any systemic hemodynamic effects and significantly decreased portal pressure and fibrogenesis^5,8,19^. Similarly, the present study demonstrates that the delivery of the drug was specific, as shown by the staining of human serum albumin, which could only derive from the carrier, in desmin-positive cells in cirrhotic rat livers, and previously demonstrated^5,8,19^. Moreover, ROCK inhibition in these cells was demonstrated by staining for p-MLC in the fibrotic septa, which represent the activated HSC, as previously described^19^. Also, by using Y27pPBHSA, no extrahepatic hemodynamic effects were observed.

This extrahepatic hemodynamic effect might be deleterious, especially since the decrease in MAP could decrease renal perfusion, as observed for different drugs (e.g. losartan)^35^, an extremely important feature in decompensated cirrhosis associated with mortality^36^. Nevertheless, using targeting strategies to M6P receptor, Y27 elicited only mild systemic hemodynamic effects, while renal perfusion also remained unaltered^19^.

Renal dysfunction and sodium/water retention are frequent in cirrhosis^37^ and are linked to development of ascites and increased short-term mortality^38^. Human data, especially, have demonstrated that a large number of cirrhotic patients have pathological features in kidney histology^39^. These clinical data were confirmed in our study, which showed that the cirrhotic kidney had increased *ngal* transcription compared to non-cirrhotic controls. Interestingly, in renal injury, but also in the presence of liver disease, PDGFRβ expression in the kidney increases^22,23^, as demonstrated in the present study by increased renal *pdgfrb* mRNA expression. This observation might indicate that kidney function is extremely important in liver disease and also that liver disease at the stage of cirrhosis might induce kidney pathology, similarly to kidney fibrosis. Moreover, our study demonstrated for the first time that ROCK overactivation is present in cirrhotic kidneys, which possibly induces contraction and reduces renal perfusion in cirrhosis. Consequently, Y27pPBHSA could have recognized the PDGFRβ in kidneys as a target and thereby increased renal perfusion in the cirrhotic rats. However, the maximum effect on renal perfusion was observed after 24 hours in the BDL animals, suggesting other effects, besides the direct effect due to ROCK inhibition, of which only a small part might have induced the increase in renal perfusion. Possibly, the observed effects are not only due to ROCK inhibition, but also to HSA delivery to the kidneys as shown by renal pPBHSA ELISA. It is known that HSA is beneficial in decompensated cirrhosis as it improves kidney function, but not renal damage. However, it is difficult to decipher whether the renal effects of Y27pPBHSA were due to renal HSA delivery or ROCK inhibition. The reasons why the renal damage was not ameliorated are basically two. On the one side the inhibitor was given for a very short time precluding a strong effect on the pathology, and on the other side the kidney damage induced during liver cirrhosis is rather a functional damage and not a structural damage. For this reason we do not expect the improvement in pathology, but a functional improvement, which was found in these experiments.

Another indication that the observed renal effects were not secondary to the improved hemodynamic is the effect of Y27pPBHSA on cardiac function. The cardiac function is of utmost importance in liver cirrhosis since an increase in cardiac output to maintain MAP, especially the remaining cardiac reserve, plays an important role in its pathophysiology. Moreover, impaired cardiac function could result in an inadequate response to stress, such as infection, and predispose towards renal dysfunction and failure^40–42^. Therefore, current drugs limiting cardiac function, e.g. non-selective β-blockers, might be deleterious in decompensated cirrhosis^43^. Interestingly, in our setting, Y27pPBHSA resulted in no major or statistically significant changes in cardiac contractility. However, a trend towards lower cardiac output after treatment persisted. The decrease in cardiac output seems not to be due to a direct effect of the drug, but rather an indirect effect due to the decreased portal pressure. Nevertheless, cardiac output was quite heterogeneous and the decrease (even though potentially beneficial) was not statistically significant, therefore great care is required in the interpretation of potential pathophysiological implications. Hence, the improved renal perfusion was not due to an increase in cardiac output or MAP, but probably a result of intrarenal effects of Y27pPBHSA.

While MAP remained stable and even slightly increased, the decrease in cardiac output, although not significant, should be carefully evaluated. However, this study does not deliver molecular evidence for a specific effect on the heart. There was no significant *rock2* mRNA overexpression in the heart in cirrhotic rats. Moreover, p-MLC/MLC ratio was reduced only in BDL cirrhotic and p-moesin/moesin only in CCl_4_ cirrhotic Y27pPBHSA-treated rats, both without reaching significance. These results indicate a minor effect of Y27pPBHSA on cardiac ROCK inhibition, probably due to heterogeneity of the cardiac expression of these proteins in BDL- and CCl_4_-induced cirrhosis. Nevertheless, this inconsistent effect was not strong enough to reduce cardiac contractility in the cirrhotic rats.

In the liver, by contrast, PDGFRβ defines and quantifies liver fibrosis. It is abundantly distributed in the cirrhotic liver and has been associated with poor outcome in HCV cirrhosis^21^. Moreover, induction of PDGFRβ in HSC is a key factor for onset and progression of hepatic injury^21^. Thus, selective PDGFRβ drug targeting allows the delivery of compounds to inhibit ROCK-mediated HSC contraction during cirrhosis, as shown by the decreased hepatic p-moesin and p-MLC in Y27pPBHSA-treated rats.

Previous publications have shown that other PDGFRβ-targeted drugs ameliorated liver fibrosis and HSC proliferation by reducing ECM deposition and proliferation of myofibroblast-like cells^20^. The benefit of the present study is that Y27pPBHSA improves acutely PTH and renal perfusion, without decreasing MAP. Further studies should evaluate the longer-term effects, repetitive use of this compound, as well as the role of interruption of the treatment on liver fibrosis and portal hypertension.

In conclusion, targeting the ROCK inhibitor Y27 to PDGFRβ decreased fibrogenesis and portal pressure without systemic off-target effects, but improved renal perfusion.

## Material and Methods

### Synthesis and characterization of Y27pPBHSA

pPBHSA was prepared as described^44^. The Universal Linkage System ULS^TM^ (Linksys Diagnostics, The Netherlands) was coupled to Y27632 through a coordinative bond by Linksys^44^. Subsequently, we conjugated this Y27-ULS to the drug carrier pPBHSA as described^5^. The resulting drug-protein conjugate was then dialyzed and the monomeric protein fraction was purified by size-exclusion chromatography. The amount of Y27632 coupled to pPBHSA was determined by MALDI-TOF mass spectrometry and HPLC as described^5^.

### *In vitro* collagen contraction assays

To test the biological activity of Y27pPBHSA, 1 × 10^4^ LX2 cells were allowed to adhere to hydrated collagen type I hydrogels (BD Biosciences, Bedford, MA, USA) present in a 24-well dish in triplicate for each group. Gels were detached from the walls of the dish and incubated in 10% FBS/DMEM, Y27 (10 μM), Y27pPBHSA (3.3 μM) or pPBHSA (3.3 μM) for 72 h. Collagen gel area was measured by Image J (NIH, Bethesda, MD, USA).

### Animal models

Fifty male Sprague-Dawley rats (100–120 g b.w.) were purchased from Charles River Laboratories International, Inc. (Wilmington, MA, USA). Cirrhosis was induced by bile duct ligation (BDL) for 4–6w or CCl_4_ exposure for 14–16w. CCl_4_-intoxicated rats received phenobarbital (3.0 mg/kg/d) in their drinking water to induce cytochrome P450 metabolic activity^45,46^. After formation of ascites, BDL- and CCl_4_-induced cirrhotic rats were i.v. injected with Y27pPBHSA (0.5 and 1 mg/kg b.w.) and analyzed after 3 h, 6 h and 24 h. Sham-operated and age-matched non-treated rats were used as healthy controls.

All experiments were performed in accordance with the German Animal Protection Law and the Guidelines of the animal care facility (Haus für experimentelle Therapie, University Clinics Bonn, Germany), and approved by the North Rhine-Westphalia State Agency for Nature, Environment, and Consumer Protection (LANUV, file reference LANUV NRW, 84-02.04.2014.A137).

### *In vivo* hemodynamic studies

Hemodynamic studies were performed on cirrhotic rats as described previously^47^. To assess the time- and dose-dependent effect of Y27pPBHSA, doses of 0.5 and 1 mg/kg b.w. were administered via the tail vein. Invasive measurements of mean arterial pressure (MAP), hepatic-vascular resistance (HVR), hepatic portal venous flow (HPVF), and portal pressure (PP) were performed in BDL and CCl_4_ cirrhotic rats 3 h, 6 h, and 24 h after treatment. Intrahepatic vascular resistance (mmHg*min/mL*100 g) was calculated as (PP/HPVF) and adjusted to 100 g b.w.

Previous studies using the same ROCK inhibitor^8^ and also using the same carrier but a different drug^48^, did show neither deleterious effects on the systemic pressure, nor accumulation of the drug after 6 injection within 2 weeks in cirrhotic mice. For this reason we did not obtained repetitive injections, but focused on two different models with a detailed hemodynamic assessment.

Rats were anesthetized with ketamine/xylazine (78 mg/kg and 10 mg/kg b.w.). The colored microsphere technique was performed as previously described^47^. Briefly, 300,000 systemic (red/white) microspheres (15 μm diameter, Triton-Technologies, San Diego, USA) were injected in the left ventricle before and after the injection of Y27pPBHSA. Mesenteric portal-systemic shunt volume was estimated before and after injection of 150,000 microspheres (yellow/blue) in the ileocecal vein. Animals were sacrificed by a lethal dose of ketamine under anesthesia.

### Histological staining

To detect the hepatic distribution of Y27pPBHSA, co-localization studies were performed on frozen liver sections immunostained with HSA and desmin (HSC marker) or collagen-I and p-MLC. Tissues were incubated overnight at 4 °C with primary antibodies. The source of commercially obtained antibodies can be found in Suppl. Table S1. After washing with PBS, livers were incubated for 2 h at RT with Alexa Fluor^®^ 594 goat anti-rabbit secondary antibody (A11072 Invitrogen, Rockford, IL, USA) and goat anti-mouse Alexa Fluor^®^488, (A11001, Invitrogen, Rockford, IL, USA).

Next, livers were washed twice with PBS 1x - Tween 0.05% and once with PBS 1x. Slides were mounted in SlowFade^®^ Gold antifade reagent with DAPI (Life Technologies, Eugene, OR, USA). Negative controls were accomplished by omitting the primary antibody and immunofluorescence staining was detected at 630x magnification.

Details on general methodology, such as Sirius red staining, αSMA immunohistochemistry (IHC), hepatic hydroxyproline content (HP) quantification, pPBHSA ELISA, and Western blotting, have been described previously^6,29,49–54^.

### qPCR

Real time PCR was developed with diluted cDNAs using pre-developed and validated TaqMan^®^ gene expression assays by Applied Biosystems or FastStart SYBR Green Master (Roche, Branchburg, NJ), according to the manufacturer´s protocol. Amplification by real-time PCR was performed on the 7300 Real-Time PCR System (Applied Biosystems, Foster City, CA, USA). Each qPCR analysis included triplicate wells and appropriated control reactions were performed in all samples. The expression of each gene of interest was calculated by the delta-delta Ct method. Gene amplification was normalized against *18s rRNA* expression in each sample and gene expression levels are shown as relative expression units compared to control group. The complete list of gene expression assays is shown in Suppl. Table S2.

### Statistical analysis

Statistical analysis was performed using Prism V.5.0 (GraphPad, San Diego, California, USA). Data are expressed as mean ± SEM unless otherwise specified. Statistical comparisons among groups were performed by two-factor analysis of variance (ANOVA). All experiments were performed in triplicate at least four times and a representative image or blot is shown in all Figures.

## Supplementary information

Supplementary Information File
