# Supplementary material for: Rho-kinase inhibitor coupled to peptide-modified albumin carrier reduces portal pressure and increases renal perfusion in cirrhotic rats
Source: Sci Rep. 2019 Feb 19;9:2256. doi: 10.1038/s41598-019-38678-5 (PMC6381202; doi:10.1038/s41598-019-38678-5)
Supplement: Supplementary file 1 — Supplementary Information File [file 41598_2019_38678_MOESM1_ESM.docx]

**Rho-kinase inhibitor coupled to peptide-modified albumin carrier reduces portal pressure and increases renal perfusion in cirrhotic rats**

Sabine Klein, Franziska Frohn, Fernando Magdaleno, Catharina Reker-Smit, Robert Schierwagen, Irela Schierwagen, Frank Erhard Uschner, Fransien van Dijk, Dieter O. Fürst, Sonja Djudjaj_,_ Peter Boor, Klaas Poelstra, Leonie Beljaars, Jonel Trebicka

**SUPPORTING INFORMATION**

**SUPPLEMENTARY TABLES**

**Table S1. List of commercially available antibodies used**

| **Target** | **Antibody** | **Source** |
| --- | --- | --- |
| Human serum albumin | VMA00071 | Bio-rad |
| Collagen-I | MA1-26771 | Invitrogen |
| Desmin | 4024S | Cell signaling |
| GAPDH | sc-25778 | Santa Cruz Biotechnology |
| MLC2 | 8505 | Cell signaling |
| Moesin | 3150 | Cell signaling |
| pMLC2 ^Thr18/Ser19^ | 3674 | Cell signaling |
| pMoesin^Thr558^ | sc-12895 | Santa Cruz Biotechnology |

**Table S2. List of gene expression assays and sequences**

| **Gene** | **TaqMan^®^ assay ID or gene sequences** |
| --- | --- |
| *acta2* | Rn01759928_g1 |
| *col1a1* | Rn00801649_g1 |
| *pdgfrb* | Rn00709573_m1 |
| *rock2* | Rn00564633_m1 |
| *ngal* | 5’-TCTGGGCCTCAAGGATAACAAC-3’  3’-AGACAGGTGGGACCTGAACCA-5’ |

| **Table S3: Hemodynamic characteristics of Y27pPBHSA-treated BDL and CCl_4_ cirrhotic rats** | | | | | | | | | | | | | | | | | | | | | | | | | | | |
| --- | --- | --- | --- | --- | --- | --- | --- | --- | --- | --- | --- | --- | --- | --- | --- | --- | --- | --- | --- | --- | --- | --- | --- | --- | --- | --- | --- |
|  |  |  |  |  |  |  |  |  |  |  |  |  |  |  |  |  |  |  |  |  |  |  |  |  |  |  |  |
| **Groups** | **Controls** | | | **1.0mg/kg Y27pPBHSA** | | | **BDL** | | | **BDL +  0.5mg/kg Y27pPBHSA (3h)** | | | **BDL + 1.0mg/kg Y27pPBHSA (3h)** | | | **BDL +  1.0mg/kg Y27pPBHSA (6h)** | | | **BDL +  1.0mg/kg Y27pPBHSA (24h)** | | | **CCl_4_** | | | **CCl_4_ +  1.0mg/kg Y27pPBHSA (3h)** | | |
| Portal pressure  (mmHg) | 7.71 | ± | 0.42 | 8.45 | ± | 0.64 | **18.75*** | ± | 0.70 | 19.26 | ± | 1.20 | **12.64**^●●^ | ± | 1.34 | 17.98 | ± | 2.46 | 19.67 | ± | 3.41 | **18.64*** | ± | 1.24 | **13.29**^●^ | ± | 0.83 |
| Hepatic-vascular-resistance (mmHg*min/ml*100g) | 3.59 | ± | 0.59 | 3.33 | ± | 0.53 | **6.44*** | ± | 1.12 | 7.02 | ± | 2.21 | **3.11**^●^ | ± | 0.30 | 6.27 | ± | 1.24 | 4.40 | ± | 1.10 | **8.36*** | ± | 0.52 | **3.61^●^** | ± | 0.38 |
| Hepatic arterial flow (ml/min*100g) | 1.04 | ± | 0.15 | 0.98 | ± | 0.47 | **5.81*** | ± | 2.22 | 4.87 | ± | 1.32 | 1.68 | ± | 0.74 | 6.46 | ± | 3.72 | 5.46 | ± | 3.15 | **2.75*** | ± | 0.89 | 1.17 | ± | 0.25 |
| Splanchnic vascular resistance (mmHg*min/ml*100g) | 19.53 | ± | 7.40 | 28.92 | ± | 9.41 | 20.59 | ± | 2.72 | 24.87 | ± | 7.58 | 30.77 | ± | 5.08 | 17.38 | ± | 4.83 | 18.44 | ± | 7.30 | 19.49 | ± | 2.79 | 15.02 | ± | 3.40 |
| Mesenteric shunt flow (ml/min*100g) | 0.00 | ± | 0.00 | 0.00 | ± | 0.00 | 1.47 | ± | 1.43 | 0.85 | ± | 0.23 | 0.40 | ± | 0.13 | 1.46 | ± | 0.65 | 2.93 | ± | 2.02 | 0.68 | ± | 0.66 | 0.15 | ± | 0.12 |
| Mean arterial pressure  (mmHg) | 97.55 | ± | 6.48 | 109.93 | ± | 8.47 | 79.07 | ± | 5.03 | 88.01 | ± | 8.73 | 68.97 | ± | 6.85 | 74.95 | ± | 6.23 | 98.81 | ± | 19.76 | 71.76 | ± | 9.54 | 75.01 | ± | 14.98 |
| Cardiac output  (ml/min*100g) | 43.43 | ± | 1.91 | 35.96 | ± | 3.12 | **33.40*** | ± | 3.56 | 29.50 | ± | 7.12 | 24.23 | ± | 2.37 | 19.43 | ± | 6.31 | 22.50 | ± | 6.26 | **25.60*** | ± | 2.28 | 18.71 | ± | 0.90 |
| Systemic vascular resistance (mmHg*min/ml*100g) | 3.09 | ± | 0.39 | 2.77 | ± | 0.95 | **1.61*** | ± | 0.25 | 3.80 | ± | 0.92 | 2.53 | ± | 0.20 | 2.83 | ± | 0.43 | 3.03 | ± | 0.72 | **1.87*** | ± | 0.27 | 2.14 | ± | 0.16 |
| Renal arterial flow (ml/min*100g) | 0.68 | ± | 0.12 | 1.63 | ± | 0.06 | 1.21 | ± | 0.21 | 1.74 | ± | 0.08 | 1.96 | ± | 1.05 | 2.54 | ± | 0.56 | **4.74**^●^ | ± | 2.27 | 1.14 | ± | 0.19 | **3.63**^●^ | ± | 1.16 |

***Table S3. Hemodynamic characteristics of Y27pPBHSA-treated cirrhotic rats*.** Data are expressed as mean ± SEM. One-way ANOVA and Mann-Whitney U test were used for comparison between groups (**p*<0.05 for cirrhotic *vs.* control animals; ^●^*p*<0.05 and ^●●^*p*<0.001 for cirrhotic Y27pPBHSA-treated *vs*. cirrhotic non-treated rats). Abbreviations: BDL, bile duct ligation.

| **Table S4: Biochemical parameters of Y27pPBHSA-treated BDL and CCl_4_ cirrhotic rats** | | | | | | | | | | | | | | | | | | | | | | | | | | | |
| --- | --- | --- | --- | --- | --- | --- | --- | --- | --- | --- | --- | --- | --- | --- | --- | --- | --- | --- | --- | --- | --- | --- | --- | --- | --- | --- | --- |
|  |  |  |  |  |  |  |  |  |  |  |  |  |  |  |  |  |  |  |  |  |  |  |  |  |  |  |  |
| **Groups** | **Controls** | | | **1.0mg/kg Y27pPBHSA** | | | **BDL** | | | **BDL +  0.5mg/kg Y27pPBHSA (3h)** | | | **BDL + 1.0mg/kg Y27pPBHSA (3h)** | | | **BDL +  1.0mg/kg Y27pPBHSA (6h)** | | | **BDL +  1.0mg/kg Y27pPBHSA (24h)** | | | **CCl_4_** | | | **CCl_4_ +  1.0mg/kg Y27pPBHSA (3h)** | | |
| **ALT (U/L)** | 50.11 | ± | 2.26 | 45.25 | ± | 1.26 | **132.36*** | ± | 29.09 | 109.75 | ± | 21.86 | 109.03 | ± | 15.80 | 115.21 | ± | 23.58 | 87.83 | ± | 17.55 | **282.92*** | ± | 80.04 | 287.00 | ± | 14.65 |
| **AST (U/L)** | 105.93 | ± | 10.09 | 109.04 | ± | 8.01 | **621.10*** | ± | 208.57 | 607.57 | ± | 132.37 | 518.08 | ± | 104.65 | 654.59 | ± | 155.87 | 487.75 | ± | 78.79 | **491.62*** | ± | 169.28 | 412.23 | ± | 68.21 |
| **Bilirubin (mg/dL)** | 0.06 | ± | 0.01 | 0.07 | ± | 0.02 | **5.39*** | ± | 1.20 | 7.41 | ± | 0.47 | 5.31 | ± | 1.15 | 6.63 | ± | 1.48 | 6.58 | ± | 0.90 | 0.30 | ± | 0.08 | 0.24 | ± | 0.06 |
| **Creatine kinase**  **(units/L)** | 2532.7 | ± | 501.1 | 1876.0 | ± | 285.9 | 2859.5 | ± | 1013.5 | 2959.9 | ± | 679.8 | 3070.0 | ± | 1122.5 | 1658.2 | ± | 506.1 | 1434.3 | ± | 289.2 | 2511.0 | ± | 383.9 | 2804.3 | ± | 921.8 |
| **Creatinine**  **(mg/dL)** | 0.52 | ± | 0.05 | 0.66 | ± | 0.02 | 0.55 | ± | 0.06 | 0.50 | ± | 0.06 | 0.58 | ± | 0.07 | 0.64 | ± | 0.11 | 0.56 | ± | 0.06 | 0.62 | ± | 0.09 | 0.66 | ± | 0.07 |
| **CRP (mg/L)** | 0.33 | ± | 0.06 | 0.53 | ± | 0.07 | 0.41 | ± | 0.17 | 0.44 | ± | 0.11 | 0.67 | ± | 0.15 | 0.29 | ± | 0.17 | 0.15 | ± | 0.03 | 0.74 | ± | 0.29 | 0.80 | ± | 0.33 |
| **Sodium**  **(mmol/L)** | 136.5 | ± | 2.0 | 135.5 | ± | 1.1 | 137.2 | ± | 1.7 | 144.8 | ± | 2.0 | 143.6 | ± | 1.4 | 140.9 | ± | 4.3 | 140.0 | ± | 2.3 | 140.6 | ± | 1.0 | 142.3 | ± | 3.2 |
| **UREA (mg/dL)** | 36.43 | ± | 3.11 | 42.27 | ± | 2.36 | 44.41 | ± | 3.57 | 61.22 | ± | 4.72 | 48.22 | ± | 4.50 | 56.34 | ± | 6.10 | 50.10 | ± | 4.19 | 34.78 | ± | 1.74 | 48.52 | ± | 7.14 |

***Table S4. Biochemical parameters of Y27pPBHSA-treated cirrhotic rats*.** Data are expressed as mean ± SEM. One-way ANOVA and Mann-Whitney U test were used for comparison between groups (**p*<0.05 for cirrhotic *vs.* control animals; ^●^*p*<0.05 and ^●●^*p*<0.001 for cirrhotic Y27pPBHSA-treated *vs*. cirrhotic non-treated rats). Abbreviations: BDL, bile duct ligation; ALT, alanine aminotransferase; AST, aspartate aminotransferase; CRP, C-reactive protein.

**SUPPLEMENTARY FIGURES**

***
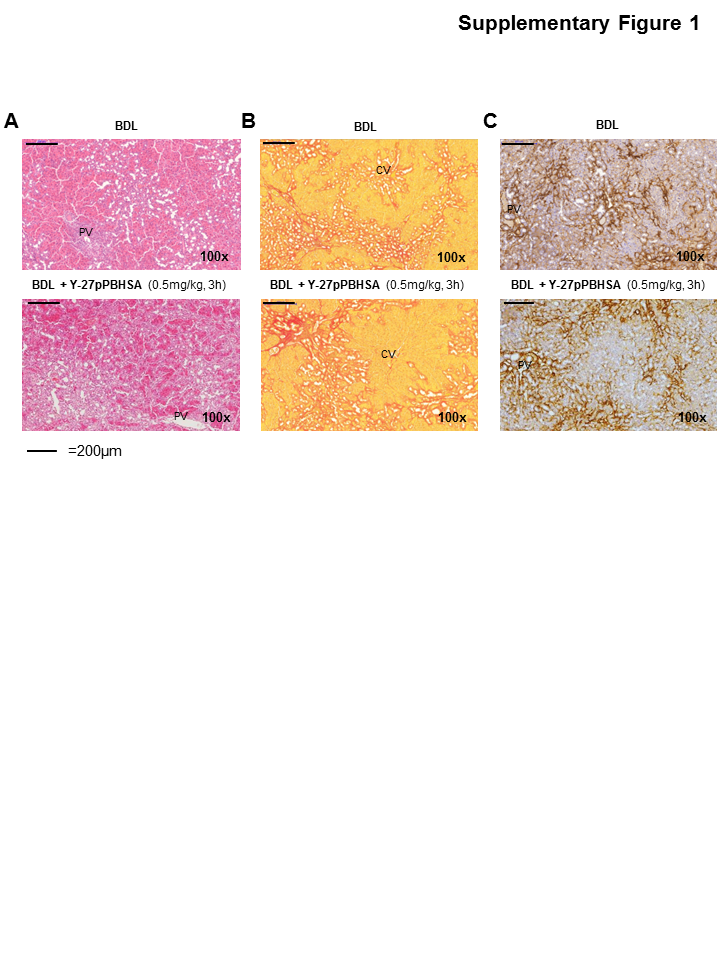
***

***Supplementary Figure 1. Y27pPBHSA-treated BDL cirrhotic rats had similar periportal injury, Sirius red staining and αSMA protein expression than non-treated cirrhotic rats*.** H&E staining **(A)** Sirius red staining **(B)** and αSMA IHC **(C)** in livers from BDL cirrhotic and Y27pPBHSA-treated BDL cirrhotic rats (0.5mg/kg) 3h after i.v. injection. Abbreviations: BDL, bile duct ligation.

***
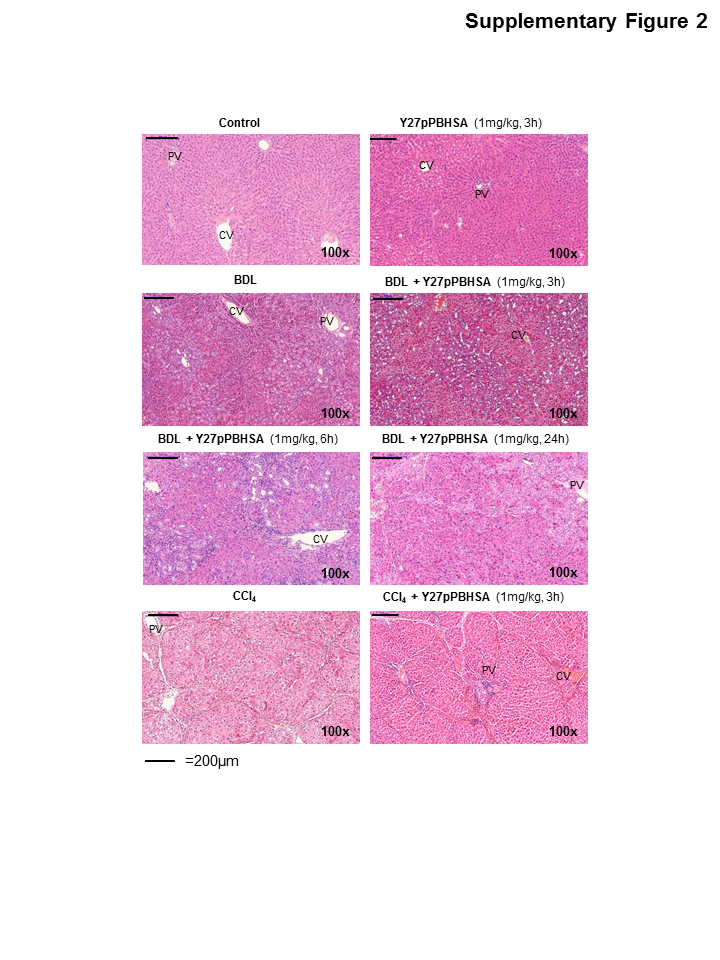
***

***Supplementary Figure 2. Y27pPBHSA-treated cirrhotic rats had similar hepatic injury than non-treated cirrhotic rats.*** H&E staining reveals similar histological injury in livers from Y27pPBHSA-treated BDL and CCl_4_ cirrhotic rats (1.0mg/kg i.v. injection) compared to non-treated cirrhotic rats. Abbreviations: BDL, bile duct ligation.


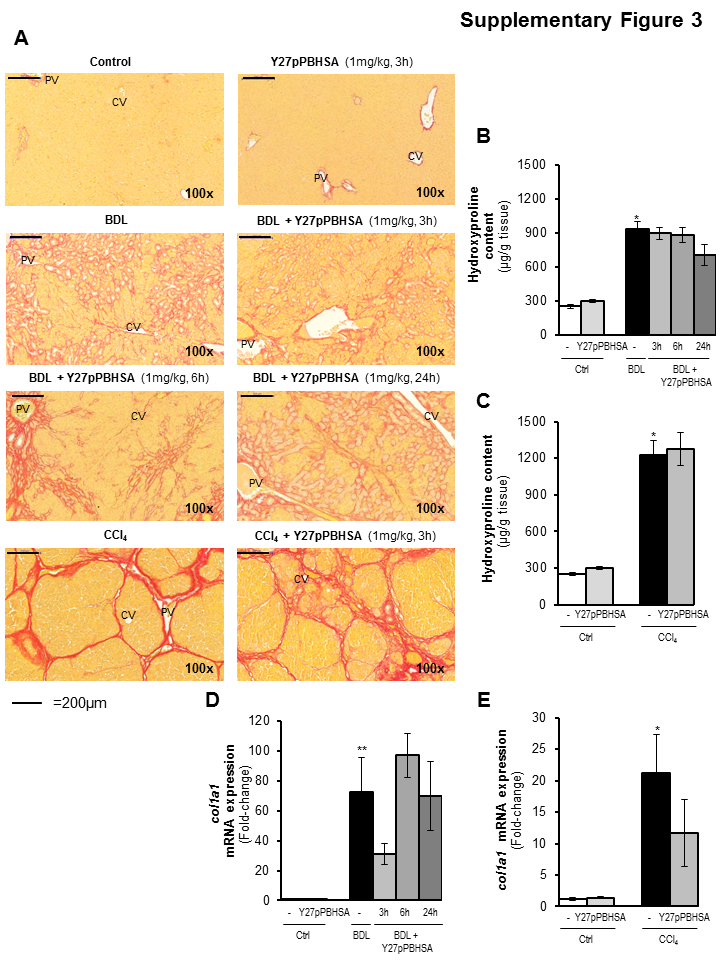


***Supplementary Figure 3. Y27pPBHSA reduces fibrogenesis in cirrhotic rats.*** Sirius red staining **(A)**, hepatic hydroxyproline content **(B-C)** and *col1a1* mRNA expression **(D-E)** in livers from BDL and CCl_4_ cirrhotic rats treated with Y27pPBHSA (1mg/kg). Results are expressed as mean ± standard error of the mean (SEM); n=6/group. **p*<0.05 and ***p*<0.01 for BDL and CCl_4_ cirrhotic *vs*. corresponding control rats. Abbreviations: BDL, bile duct ligation.


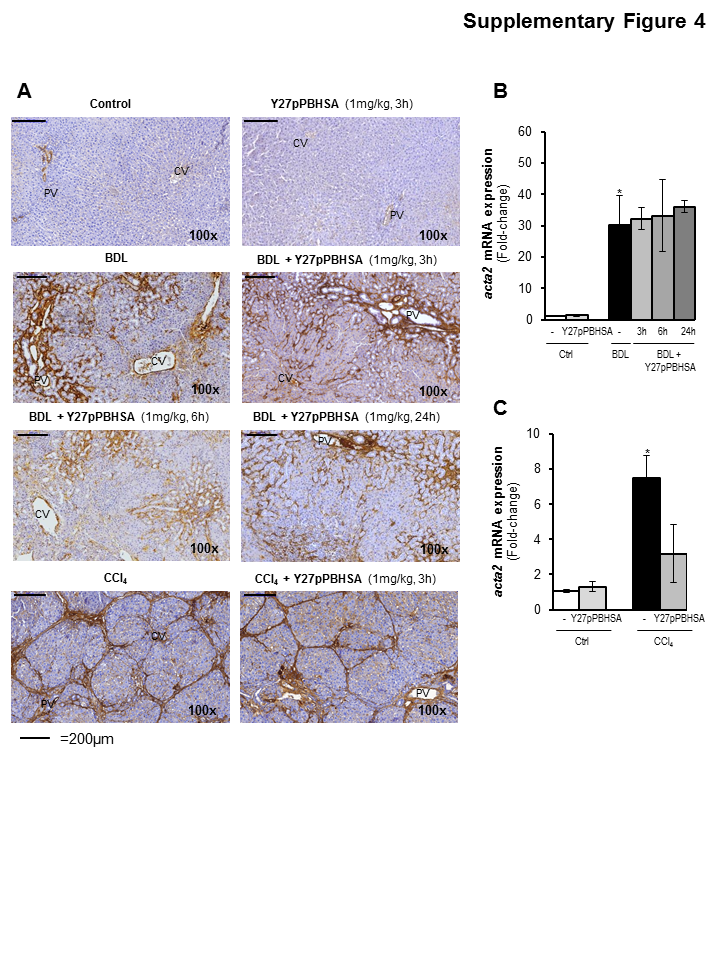


***Supplementary Figure 4. Y27pPBHSA-treated rats have similar hepatic αSMA and acta2 mRNA expression compared to cirrhotic rats****.* αSMA IHC **(A)** and hepatic *acta2* mRNA expression from Y27pPBHSA-treated BDL and CCl_4_ cirrhotic rats **(B-C).** Results are expressed as mean ± standard error of the mean (SEM); n=6/group. **p*<0.05 for BDL or CCl_4_ cirrhotic *vs*. corresponding control rats. Abbreviations: BDL, bile duct ligation.


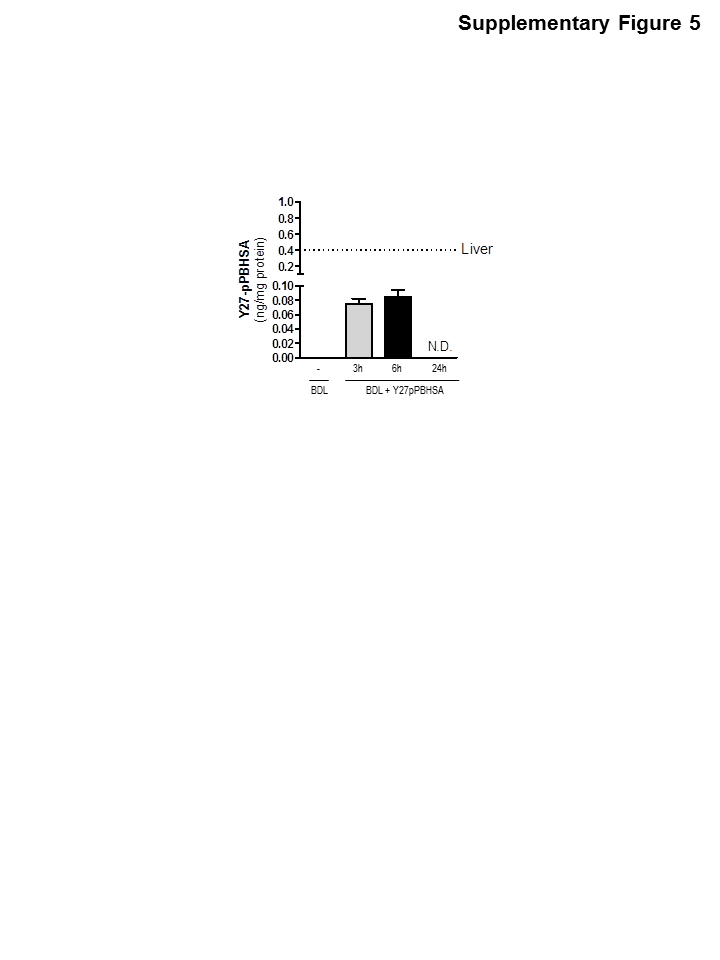


***Supplementary Figure 5. Y27pPBHSA-treated rats have trace amounts of Y27pPBHSA in the kidney.*** pPB-HSA ELISA in kidneys from BDL-cirrhotic rats (ng/mg kidney-protein). Results are expressed as mean ± standard error of the mean (SEM); n=3/group. Abbreviations: BDL, bile duct ligation; ND, not detected.
